# Performance of hybrid Innegra-carbon fiber composites

**DOI:** 10.1038/s41598-023-47353-9

**Published:** 2023-11-27

**Authors:** Uday Vaidya, Balaji Thattaiparthasarthy, Mark Janney, Mark Mauhar, Keith Graham, Elizabeth Cates

**Affiliations:** 1 Tickle College of Engineering, University of Tennessee, 1512 Middle Drive, Knoxville, TN 37996 USA; 2 Manufacturing Sciences Division (MSD), Oak Ridge National Laboratory (ORNL), 2350 Cherahala Blvd, Knoxville, TN 37932 USA; 3 The Institute for Advanced Composites Manufacturing Innovation, 2370 Cherahala Blvd, Knoxville, TN 37932 USA; 4 Materials Science & Engineering, University of Alabama at Birmingham, 1150 10th Avenue South, Birmingham, USA; 5 Carbon Conversions, Incorporated, Lake City, SC USA; 6 Innegra Technologies, Greenville, SC USA

**Keywords:** Engineering, Materials science

## Abstract

In this work material synergy with high stiffness carbon fiber with ductile high strength polypropylene fiber (Innegra S), (referred to as Innegra, herein) have been evaluated in a range of laminate designs. Both woven and discontinuous carbon fiber have been considered. The discontinuous fibers are based on three-dimensional deposition (3DEP) (referred to as 3DEP, herein) carbon fiber preform process. Eleven (11) variants of Innegra-carbon fiber hybrid laminates were investigated for tensile, flexure, compression, in-plane shear and low velocity impact response. The effect of position of Innegra within the laminate was studied and found to influence strength and stiffness properties. In terms of overall trends, Innegra provides upward of 18% improvement in strain (ductility) to the composite and eliminates brittle fracture of carbon fiber. The moduli trends follow the proportionality of Innegra fiber to carbon fiber plies. However, the strength is controlled by the interface between Innegra and carbon fiber. The primary failure mode in tension and compression is via onset of debonding between Innegra and carbon fiber. The 3DEP carbon fiber constituents provided highest values of in-plane shear indicating that three-dimensional (3D) network of carbon fiber provides higher shear resistance. The Innegra intensive variants exhibited superior energy absorption under low velocity impact, at energy levels 15–60 J. This work provides insight for designers to incorporate Innegra and carbon fiber hybrids in composite structures.

## Introduction

Innegra S (Innegra) is a polypropylene based fiber with superior impact energy absorption and lightweight attributes^1^. When high modulus fibers are combined (hybridized) with Innegra fiber, the overall weight can be reduced, impact resistance, damage tolerance and signal transmission improved^1–3^. Hybridizing polypropylene fibers offers high strain and energy absorption, while carbon fiber provides high stiffness^3–6^. The hybridization of Innegra with carbon fiber is expected to prevent shatter of carbon fiber composites. The Innegra fiber also provides high damping and energy dissipation^4, 5^.

Hybridization of Innegra-carbon fiber can be considered in different ways, for e.g., by (a) intra-ply, interwoven carbon fiber (fabric) and Innegra tows/yarns, or (b) inter-ply, discrete layering of Innegra and carbon fiber (fabric)^7, 8^. HIA Velo bicycle company features carbon/Innegra in strategic areas of the Alfa Road bike frame^5^. Battery operated carbon fiber surfboards have used carbon/Innegra prepreg expanded polystyrene (EPS) sandwich composite^1^. Hybrids composites have also been evaluated by combining Innegra, Kevlar, basalt, E-glass, S-glass, and carbon fiber^7, 8^.

## Literature review

Several researchers have successfully used hybridization to enhance mechanical properties and improve damage resistance of composites^9–15^. Hybridization in woven fabric composites offers benefits due to the synergy of interlacing fiber tows in different directions. Hosur et al.^9^ investigated low velocity impact (LVI) of hybrid laminates of twill weave carbon fabric and plain weave S2-glass fabric composites. They observed considerable improvement in the load carrying capability of hybrid composites as compared to carbon/epoxy laminates, with only slight reduction in stiffness. Park and Jang^10^ studied the effects of intra-ply hybridization on the mechanical performance of aramid/polyethylene fabric composites. They reported increased flexural strength in proportion to aramid fiber content and lower interlaminar shear strength when compared to pure polyethylene fiber composites. Thanomslip and Hogg^11^ investigated penetration impact resistance of hybrid composites based on commingled yarn fabrics of E-glass fibers and thermoplastic fibers. They obtained significant increase in the total absorbed energy with hybrid composites as compared to glass fiber composites. They concluded that plastic deformation in the thermoplastic fibers was the key factor in the improvement in energy absorption of hybrid composites. Lee et al.^12^ investigated the response of hybrid graphite and Kevlar composites. The observed that graphite–Kevlar–graphite laminate had low-energy absorption than Kevlar–graphite–Kevlar composite. Cheon et al.^13^ studied impact and interlaminar shear properties of glass fiber epoxy system hybridized with polyethylene and polypropylene fabric, and non-silane treated glass fibers and Kevlar fibers. They varied the placement of the embedded materials. They obtained 80% higher impact energy absorption with ~ 4% volume fraction of Kevlar-29 fiber and 40% increase with 5% volume fraction of non-silane treated glass fibers as compared to that of glass epoxy composite. Naik et al.^14^ investigated impact behavior and post impact compressive characteristics of glass-carbon hybrid composites with alternate stacking sequences. They concluded that hybrid composites are less notch sensitive when compared to only carbon or only glass composites. Also, carbon-outside/glass-inside clustered hybrid configuration gave lower notch sensitivity compared to other hybrid configurations. Song^15^ investigated bending behavior of carbon/glass and carbon/aramid hybrid composites. Swolfs et al.^16^ studied the bonding in intralayer carbon fiber/self-reinforced polypropylene hybrid composites for different levels of weak and strong bonding. They showed that intralayer carbon fiber/self-reinforced hybrid composites have the potential to offer an unique combination of stiffness and ultimate failure strain combined with high penetration impact resistance. Recycled carbon fibers are providing value-add to replace virgin carbon fiber^17–19^. In this work, the 3DEP process uses recycled carbon fibers to convert them into non-woven mats^20^.

In the present work, eleven (11) variants (20 layers in each variant) with different combinations of Innegra and carbon fiber were studied. Extensive tensile, compression, flexure, in-plane shear and low velocity impact tests were conducted for each of these variants. This paper provides a comprehensive study of mechanical properties for these loading conditions along with understanding of the onset and progression of failure. The results would benefit composite designers considering Innegra in conjunction with carbon fiber composites for static and dynamic performance.

## Materials and methods

**Figure 1:**
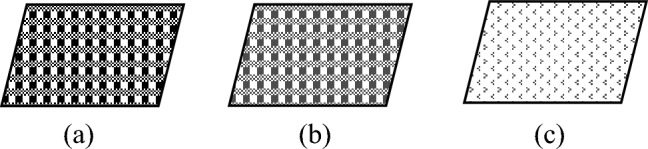
Constituents of the hybrid laminates. (**a**) Woven carbon fabric; (**b**) Woven innegra fabric, and (**c**) 3DEP discontinuous carbon fiber. Sequence of layup is shown in Table 2.

Table 1 summarizes the materials used in this study. Table 2 provides the nomenclature for the variants. The materials comprise Innegra S, 12 K woven carbon fabric and 3DEP^20, 21^ carbon fibers respectively arranged in various sequences outlined in Table 3, and Fig. 1. All laminates comprising 20 plies in each case were produced via compression molding in a two- cavity mold at 100 psi and 300 °F nominal temperature. Processing was conducted on a 30 metric ton Wabash Carver press equipped with a 275 mm × 275 mm (11″ × 11″) mold. The layup details are provided in Tables 1 and 2.

**Table 1:** Details of the materials makeup.

Constituent	Notes
Matrix	Gurit Prime 20LV epoxy cure at 60 °C for 30 min
Reinforcements	Innegra S; 1250 denier, dtex 1389, untwisted, 123 gsm
	12 K plain weave T300 standard modulus carbon fiber, 7 μm filament diameter; Balanced laminate, `200 gsm areal density
	3DEP carbon fiber, 12.7 mm (1/2″) fiber length, T300, recycled from T300 uniaxial tape, 7 μm filament diameter

**Table 2:** Nomenclature and constituents used in the variants for this study.

Sequence	Nomenclature	Woven carbon	Woven Innegra	Woven carbon & Innegra	3DEP	
					% Carbon	% Innegra
1	20C	20	0	0	N/A	N/A
2	2IC	18	2	0	N/A	N/A
3	4IC	16	4	0	N/A	N/A
4	6IC	8	6	0	N/A	N/A
5	2IS	16	2	0	N/A	N/A
6	4HC	16	0	4	N/A	N/A
7	8HC	12	0	8	N/A	N/A
8	4HS	16	0	4	N/A	N/A
9	3D/10	16	0	0	10	90
10	3D/100	8	0	0	100	N/A
11	3D/67	8	0	0	67	33

**Table 3:** Detailed layup sequence for each variant, explanation and rationale for the choice of the laminate sequence.

Sequence	Nomenclature	Architecture	Explanation	Rationale/hypothesis
1	20C	C–C–C–C–C–C–C–C–C–C–C–C–C–C–C–C–C–C–C–C	20 All carbon layers; No Innegra	Baseline
2	2IC	C–C–C–C–C–C–C–C–I–I–C–C–C–C–C–C–C–C	Two (2) Innegra layers in the middle, 8 layers of carbon on either side	Does adding two layers of Innegra affect the ductility synergy
3	4IC	C–C–C–C–C–C–I–I–I–I–C–C–C–C–C–C	Four (4) Innegra layers in the middle, 8 layers of carbon on either side	Will additional Innegra layers provide ductility without compromising flexural stiffness
4	6IC	C–C–C–C–I–I–I–I–I–I–C–C–C–C	Six (6) Innegra layers in the middle, 8 layers of carbon on either side	Will further increase ductility without compromising flexural stiffness
5	2IS	C–C–I–C–C–C–C–C–C–C–C–C–C–C–C–I–C–C	Two (2) Innegra layers, one each to the outer side, 8 layers of carbon on either side	Does position of the Innegra layers influence the performance?
6	4HC	C–C–C–C–C–C–C–C–H–H–H–H–C–C–C–C–C–C–C–C	Four (4) hybrid woven Innegra-carbon layers in the middle, 8 layers of carbon on either side	Does hybrid interwoven Innegra-carbon layers perform differently than constituent layered Innegra-Carbon
7	8HC	C–C–C–C–C–C–H–H–H–H–H–H–H–H–C–C–C–C–C–C	Eight (8) hybrid woven Innegra-carbon layers in the middle, 8 layers of carbon on either side	Does hybrid interwoven Innegra-carbon layers preform differently than constituent layered Innegra-Carbon
8	4HS	C–C–H–H–C–C–C–C–C–C–C–C–C–C–C–C–H–H–C–C	Four (4) hybrid woven Innegra-carbon layers two each toward the outside, 8 layers of carbon on inner side	Does hybrid interwoven Innegra-carbon layers preform differently than constituent layered Innegra-Carbon layers and what is its influence in terms of position (center versus towards the outer sides)
9	3D/10	C–C–C–C–C–C–C–C–[3D/10]–[3D/10]–C–C–C–C–C–C–C–C	Two layers of 3D/10% C-90% Innegra in the middle, 8 layers of carbon on either side	Does discontinuous carbon core with innegra behave differently than continuous Innegra or continuous carbon; Effect of percentage of carbon
10	3D/100	C–C–C–C–[3D/100]–[3D/100]–C–C–C–C	Two layers of 3D/100% carbon, (no Innegra) in the middle, 4 layers of carbon on either side	Does discontinuous carbon core behave differently than continuous carbon as core; Effect of percentage of carbon
11	3D/67	C–C–C–C–[3D/67]–[3D/67]–C–C–C–C	Two layers of 3D/67% carbon, (33% Innegra) in the middle, 4 layers of carbon on either side	Does discontinuous carbon core with innegra behave differently than continuous Innegra or continuous carbon; Effect of percentage of carbon

### Innegra S

Innegra S is an olefinic fiber with 90% and greater content polypropylene of 0.84 g/cc density^1^. Innegra is used with high modulus carbon fibers to increase toughness and durability minimizing catastrophic failure. The fiber provides energy dissipation, vibration damping and weight reduction attributes^4–6^. In this work a 1250 denier, dtex 1389, untwisted Innegra S fiber was used with an elastic modulus ~ 14 GPa. Innegra fiber was hybridized with carbon fiber in this work. The hybrid Innegra is 2450 dTex with Innegra fiber fraction by volume of 15–33% in case of hybrid Innegra-carbon layers.

### Woven carbon fabric

12 K plain weave carbon fiber, standard modulus T300 was used for the carbon layers^22^.

### 3DEP

MIT-RCF (now CCI) developed an innovative method for making fiber preforms^20^. The 3DEP process lends itself to converting loose recycled fibers into non-woven carbon fiber mats. The 3DEP process uses advanced slurry molding process for creating non-woven recycled carbon fiber preforms. 3DEP produces homogeneous fiber distribution within the mat with consistent areal weight and acceptable dimensional tolerance. In this work 3DEP is used to produce recycled carbon fiber mats, which are then hybridized with Innegra fibers to obtain the variants 9–11 identified in Table 2. The 3DEP process in this study used recycled carbon fiber obtained from pyrolysis of T300 uniaxial tape. The recycled fiber had nominal 1/2” (12.7 mm) fiber length and ~ 7 μm diameter^18–20^.

### Specimen design approach/rationale

The goal of the study was to evaluate the synergy of ductile high strain Innegra with high stiffness low strain carbon fiber, both in woven form and in discontinuous 3DEP form. The effect of intra-ply versus inter-ply hybridization was also studied. Further, it was of interest to evaluate the position of the Innegra layers within the laminate. The design of experiment comprised eleven (11) variants as outlined in Table 3. The rationale for the choice of the variants is also provided in this Table.

### Mechanical testing

Extensive mechanical testing was conducted including tensile, flexure, compression, interlaminar shear and low velocity impact respectively. All mechanical tests were conducted on a MTS 810 load frame with the respective fixture. The tensile and compression tests were instrumented with an extensometer. The contact of the extensometer with respect to the specimen was lost at high loads, however the cross-head displacement was recorded from the beginning to the end of the test. It may be noted that the slope was corrected to account for machine compliance when extensometer contact was lost. Low velocity impact (LVI) tests were conducted with an instrumented drop weight tower, Dynatup 8250 as discussed in the LVI section. Detailed tabulated results from tension, compression, flexure, in-plane shear and LVI are included in Appendix A (Supplemental information). These are given a prefix of Axxx meaning Appendix followed by the number.

#### Tension

ASTM D3039 was adopted for tension testing. Rectangular specimens 254 mm × 25.4 mm × 4 mm (10″ × 1″ × 0.15″) (flat wise tension) were adopted. Specimens were tabbed using woven glass/epoxy tabs. Five specimens were tested for each variant. The tests were conducted in displacement control mode at a rate of 2 mm/min. Strain was measured until complete failure using 50.8 mm (2″) gage clip on extensometer. The chord modulus calculated between 1 to 3 milli-strain. Since the failure was progressive in most samples, the extensometers had some contact issues during progressive failure, however this did not affect the calculation for the modulus.

#### Flexure

Flexural strength and modulus were measured by ASTM D790. A length divided by thickness (l/t) ratio of 16 and w/t of 0.25 was adopted. The overall length to thickness was 22 including overhang, five specimens per variant were tested under displacement control rate 1.5 mm/min. Strain measurements included ε = 6 × D × t/l^2^, where D is the maximum deflection at the center of the beam, t is the thickness of the specimen, l is the support span. The chord modulus was calculated between 1 to 3 milli-strain.

#### Compression

ASTM D6641 Compressive properties of polymer matrix composite materials using a combined loading compression (CLC) Test Fixture was used to determine the compressive properties. Rectangular specimens (lengthwise compression) 139.7 mm (5.5″) length × 012.7 mm (0.5″) width × thickness was used. Specimens were machined flat to within (0.254 mm (0.01″) along short edge. Five specimens were tested in each variant. 3 specimens were strain gaged back-to-back along the length to determine bending. Bending values over 10% were noted in the chord modulus column for each. Bending values less than or equal to 10% were not noted. The testing was conducted under displacement control at a rate of 1.4 mm/min.

#### In-plane shear

ASTM D3518 Standard test method for in-plane shear response of polymer matrix composite materials by tensile test of ± 45° Laminate was used to measure in-plane shear properties. Rectangular specimens (flat wise tension/in-plane shear) 254 mm (10″) × 25.4 mm (1″) × thickness was used. Samples were tabbed using woven glass/epoxy. Five (5) specimens were tested in each variant. Strain was measured using strain gauges in longitudinal and transverse direction. Poisson’s ratio was calculated by the difference between the longitudinal and transverse strains. Shear modulus was calculated using the Poisson’s ratio and shear strength. The test was conducted under displacement control at a rate of 2 mm/min.

#### Low velocity impact (LVI)

LVI was conducted on an Instron Dynatup 8250 drop weight impactor. Simply supported plate specimens of nominal size 100 mm × 100 mm (4″ × 4″) were used to conduct the LVI study. A 15.8 mm (5/8″) diameter hemispherical shape impactor with an impact mass of 6.15 kg (13.6 lb.) was used. Two impact energies were used namely- low impact of 15 J and high impact energy of 60 J. The force–time-energy was determined from the contact force of the impactor. Electronic flags measure the impact velocity as the light path is interrupted with the passage of the impactor.

## Results and discussion

### Tensile testing

**Figure 2:**
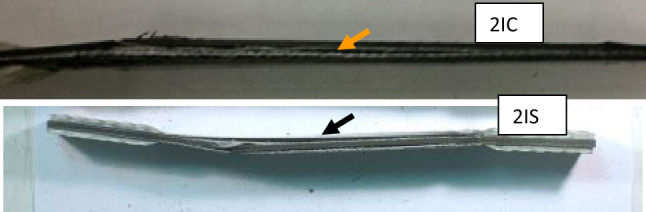
Tensile failure of 2IC versus 2IS. In both cases, the primary mode of failure is by debond initiation between Innegra and carbon fiber layers. The arrows indicates Innegra-carbon fiber layer interface sites for onset and progression of failure. Specimen size 254 mm × 25 mm × 4 mm.

**Figure 3:**
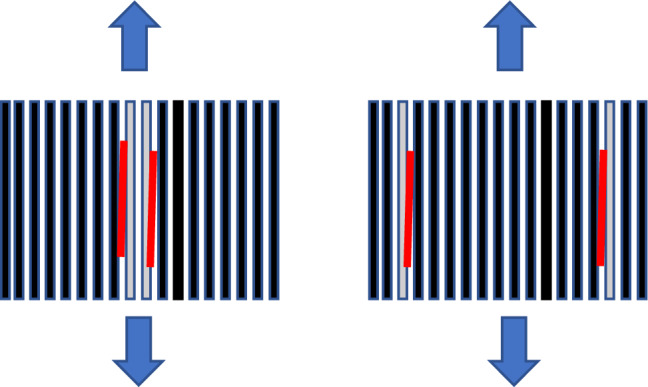
Initiation and progression of failure for the 2IC and 2IS respectively. The red lines are the origin and progression of failure, primarily along the Innegra-carbon layer interfaces.

**Figure 4:**
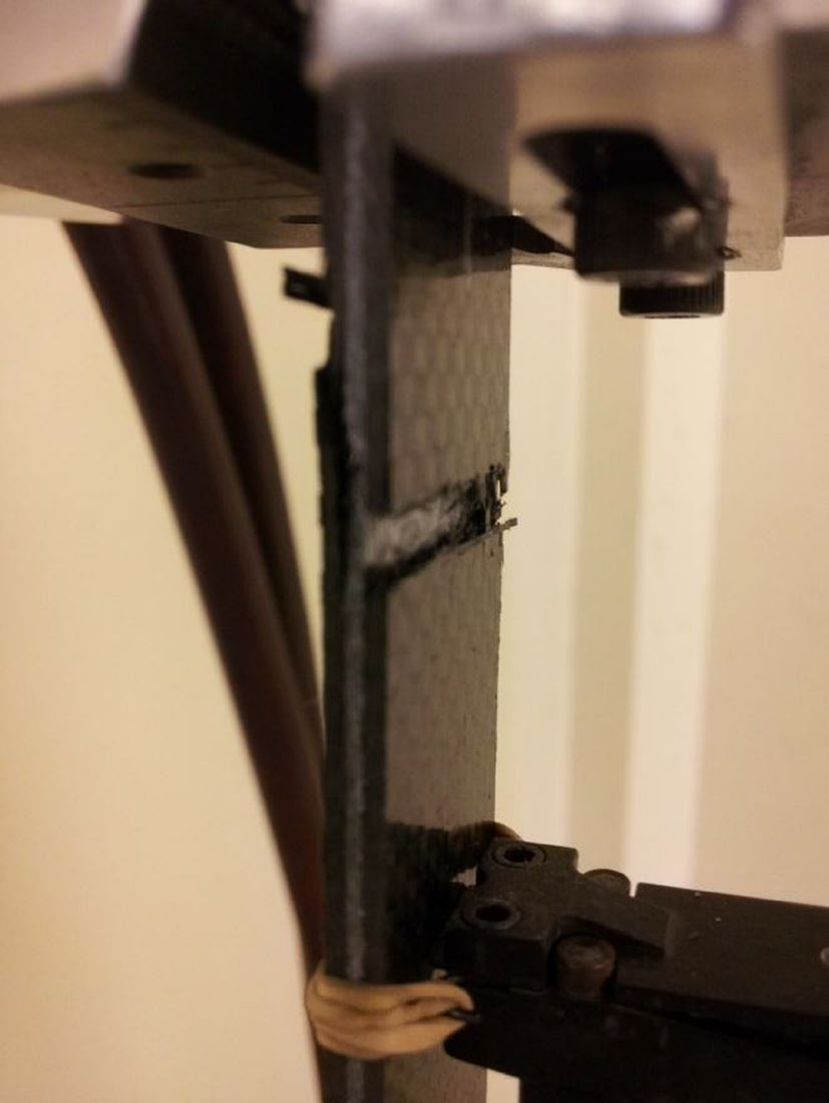
Failure of 3D/67 under tensile loading, Specimen size 254 mm × 25 mm × 4 mm.

**Figure 5:**
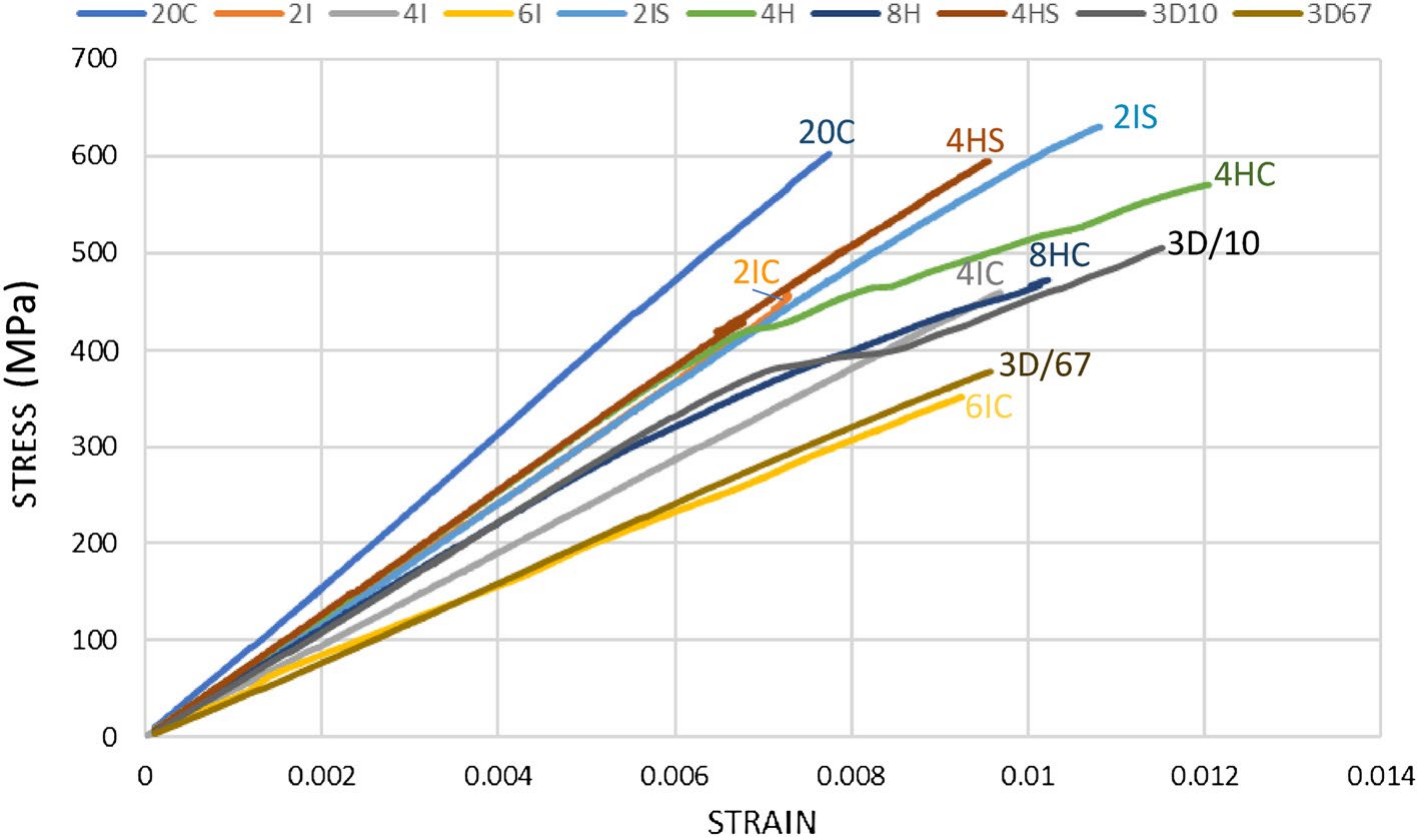
Comprehensive stress–strain curves for tensile response of different variants.

Figure 2 shows typical debonding of Innegra and carbon interfaces for Innegra-carbon hybrids 2IC and 2IS respectively. The origin and progression of failure was along the Innegra-carbon layer interface. Figure 3 illustrates the onset and progression of failure. Figure 4 illustrates brittle failure of the 3DEP–3D/67 specimen. This failure mode is in contrast to the ductile failure for 2IC and 2IS respectively. Figure 5 provides comprehensive results of the tensile stress–strain curves of all the variants from Table 3. Table A1 summarizes the average tensile strength and modulus from five specimens within each variant. Extensive data was similarly generated for all the variants and has been included in Appendix A. The following information is discussed with reference to Table A1 and Fig. 5 respectively.

### Effect of adding Innegra layers

Across all the Innegra variants, the strain within a comparable region was at least 18% or greater than 20C (all carbon). Since the strain data was truncated with respect to the extensometer, we provide the overall trend as opposed to variant-wise comparison.

#### 2IC

From Table A1 it can be noted that adding two layers of Innegra to carbon fiber (variant 2I) reduced the tensile modulus by 18% (76.9 GPa to 62.40 GPa) and tensile strength by 19% (642 MPa to 518 MPa).

#### 4IC

The tensile modulus was ~ 40% lower and ~ 25% lower strength than 20C.

#### 6IC

The tensile modulus was 56% lower, and the tensile strength was 46% lower, but the strain increases by 19%.

### Effect of ply position

#### 2IC versus 2IS

The tensile modulus for the 2IC was comparable to the 2IS (~ 60 GPa), however the tensile strength of the 2IS was 17% higher than the 2IC. The strength is clearly influenced by the location of the Innegra, i.e. performing higher when the Innegra is separated from each other, and placed closer to the outer layers of the layup. The onset and progression of failure is illustrated in Fig. 3.

#### 4HS versus 4HC

The above trend was also observed for the hybrid variants namely 4HS and 4HC. The 4HS and 4HC showed the second highest tensile modulus (65 GPa) in comparison to 20C (all carbon), 65 GPa is only about 15% lower than the 76 GPa observed for 20C. Interestingly the tensile strength of the 4HS was only 3.74% lower than the 20C. However, the tensile strength of 4HC was 49% lower than 20C and tensile strength of 4HC was 47% lower than 4HS. This is a dramatic difference for the same layup, just with different positioning of the Innegra, i.e. towards the *outer side* compared to the *center*. For 8HC the modulus was 24% lower than 20C and strength was 14% lower. We did not have a variant 8HS to compare to, but the trends suggest that separation of Innegra plies, and locating them to the outer side is more preferred to attain higher tensile strength.

#### Discussion

The lower tensile strength of the *2IC versus 2IS* and the *4HC versus 4HS* can be explained this way. It was observed that the primary mode of failure in tension is onset of debonding of the Innegra layer(s) from the carbon layers. When two or more layers of Innegra are adjacent to each other their collective stiffness causes higher extent of differential strain (constraining effect of carbon on Innegra). The weak interfacial strength of polypropylene to carbon/epoxy has been reported in^16^. The constraint offered by the carbon fiber to the Innegra plies leads to onset and progression of debonding. When the Innegra layers are separate from each other like in 2IS or 4HS, the carbon layers would offer less constraint on the Innegra (less stiff since it is a single layer), reducing the influence of differential strain between the Innegra and carbon fiber. Still the failure initiates by debonding between Innegra and carbon, and progression of debonding, albeit this occurs at a higher load level.

#### Effect of 3DEP

The three variants for the 3DEP included 10%, 100% and 67% 3DEP carbon fiber. There was less of a clear trend for the 3DEP variants. The tensile modulus for 3D/10 was 24.3% lower; 3DEP/100 was 33.8% lower and 3D/67 were 43% lower when compared to 20C. The tensile strengths were 9.6% (3D/10), 29% (3D/100) and 31% (3D/67) lower when compared to 20C respectively. Within the 3DEP variants, the 3D/100 had 12% lower strength and 3D/67 had 34% lower tensile modulus than 3D/10. The tensile strength was 27% lower for 3D/100 and 32% lower for 3D/67 compared to 3D-10. Typical failure of 3D/67 is shown in Fig. 4.

#### Discussion

It was hypothesized that 3D/100 and 3D/67 would provide higher tensile modulus and tensile strength due to high carbon fiber content. However, that was not the case. The 3D/10 (lowest carbon content, high Innegra content) had high tensile strength and modulus within the 3DEP categories. The high 3DEP carbon fiber content variants (3D/100 and 3D/67 respectively) performed lower than the high Innegra fiber content (3D/10). The interfacial strength does not seem to be fully realized. This further suggests that the three-dimensional network of entangled discontinuous carbon fibers with higher percent of Innegra fiber provides a tortuous failure path. In the inter-ply variants (such as 2IC, 4IC etc.) the interfacial debonding was the onset failure mechanism, see Fig. 4. However, that was not the case for the 3DEP variants. Microcracking in the discontinuous carbon fiber domain and the randomness associated with fiber orientation causes the onset of failure.

All the 3DEP variants were lower in values than 20C which is to be expected. 20C comprises all continuos carbon fibers, 3DEP variants comprise discontinuous carbon fibers.

### Flexural testing

**Figure 6:**
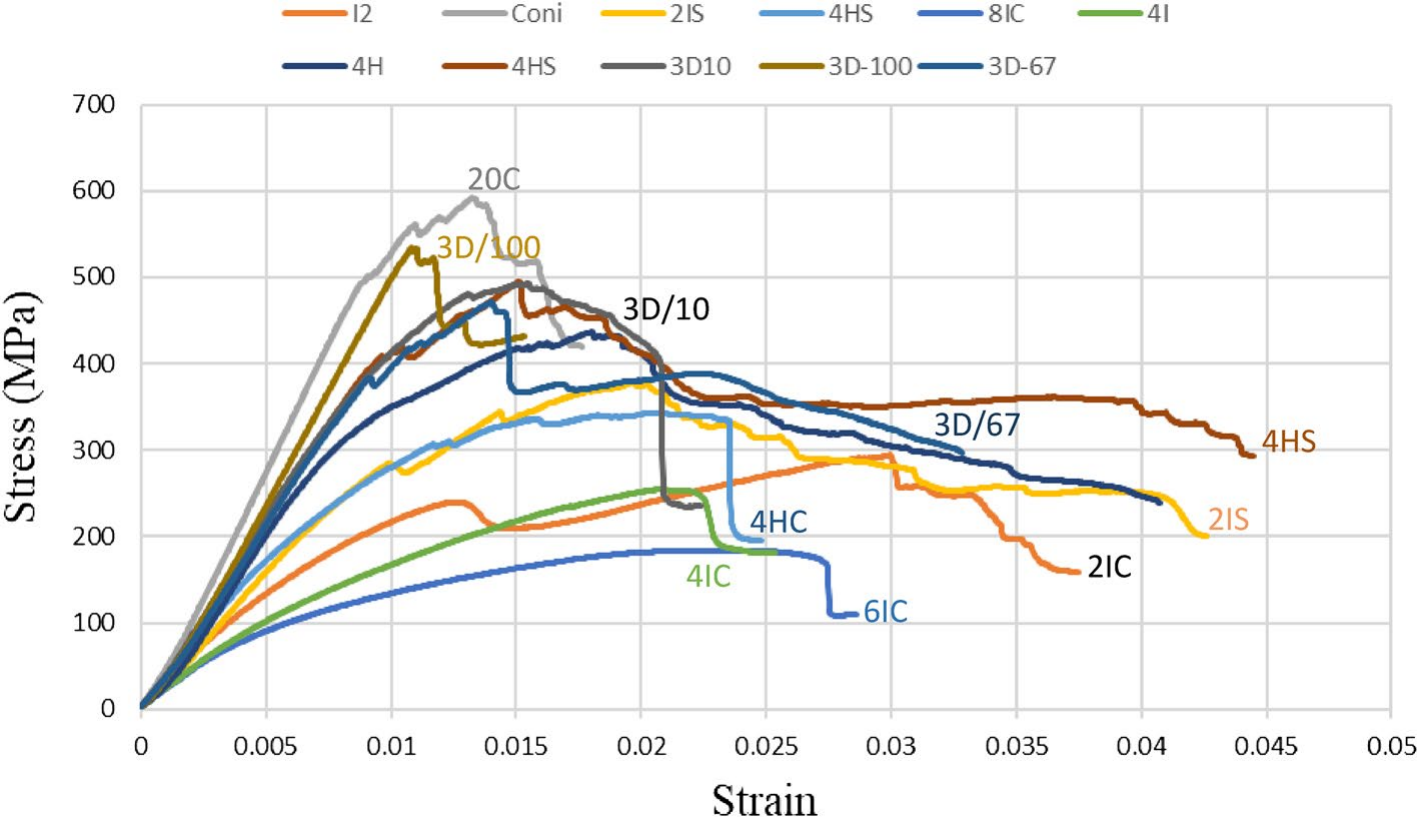
Flexural stress–strain curves for different variants.

Table A2 and Fig. 6 provide comprehensive stress-train curves for the variants under flexure.

For all variants the flexural strain increased by an average 70% in comparison to 20C indicating high degree of compliance and ability to absorb energy.

#### Effect of Innegra layers (compared to 20C) (Intra-ply)

2IC: 49% lower flexural modulus and 11.5% lower flexural strength.

4IC: 60% lower flexural modulus and 59% lower flexural strength.

6IC: 61% lower flexural modulus and 66% lower flexural strength.

#### Effect of position of the Innegra

2IC versus 2IS (Inter-ply): 22% increase in flexural modulus for 2IS versus 2IC. Flexural strength decreased 31% for 2IS versus 2IC.

4HS versus 4HC (Intra-ply): 26% and 35% decrease in flexural stress, 18% and 22% lower flexural modulus.

#### Discussion

**Figure 7:**
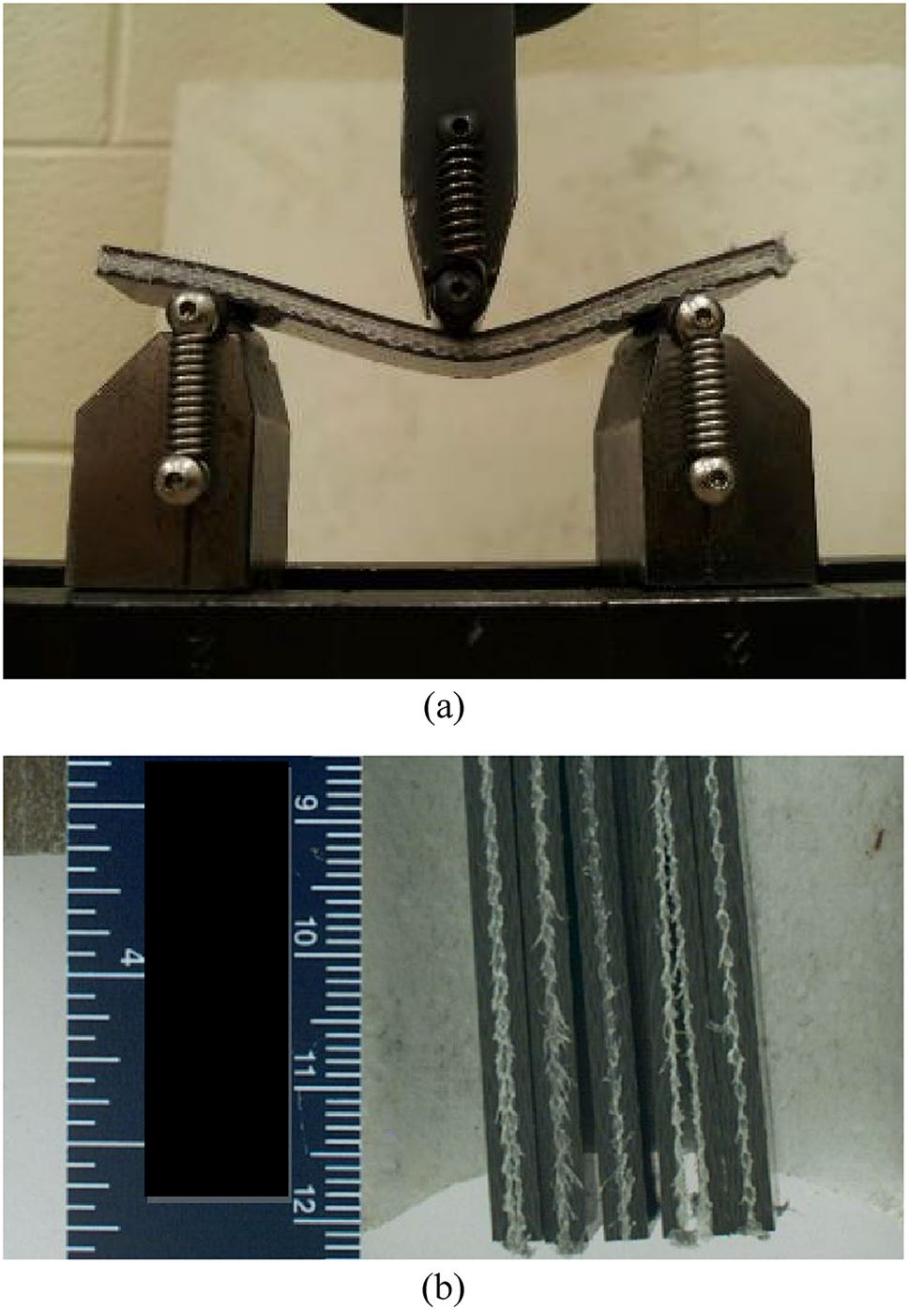
Flexural response of (**a**) Innegra 8IC illustrates high ductility; (**b**) 2IC onset and propagation of failure at the Innegra-carbon layer interface.

The Innegra variants exhibited high ductility in flexure as seen from Fig. 7a for the 8IC. This type of deformation was typically seen for all the Innegra variants and upon unloading the specimens recovered to their original flat state (hence only representative images are shown). For variants such as the 2IC, the failure was from debonding of the Innegra from the carbon interface, see Fig. 7b. In the 2IS configuration, the Innegra-carbon layer interface is in the proximity of the loading nose on the compressive side (during flexure). As seen, its modulus is 22% higher than the 2IC. The higher stiffness of the 2IS hence restricts the Innegra from bending, while promoting the onset and progression of debond at the interface between the Innegra and carbon near the compression side (loading nose). Figure 6 provides more insight into the flexural response of each variant. The 20C baseline exhibited the highest modulus as could be expected, being all carbon. The failure was progressive in all the variants, except in the 20C and 3DEP 3D/100 where it resembled brittle fracture typical of carbon fiber response. The benefits of Innegra to prevent catastrophic damage is evident from these tests with the corresponding reduction in strength and modulus.

The length to thickness (l/t) ratio of the flexural beams used in the study were 22. While the ratio of 16 up to 32 is adopted for flexural testing of composites, this study used 22. Also as seen from Tables 2 and 3 some of the layups had a sandwich like structure with the compliant material in the middle (such as 6IC). There could be marginal contribution of transverse shear in conjunction with flexural loads, however the failure modes were dominated by flexure.

### Compression testing

**Figure 8:**
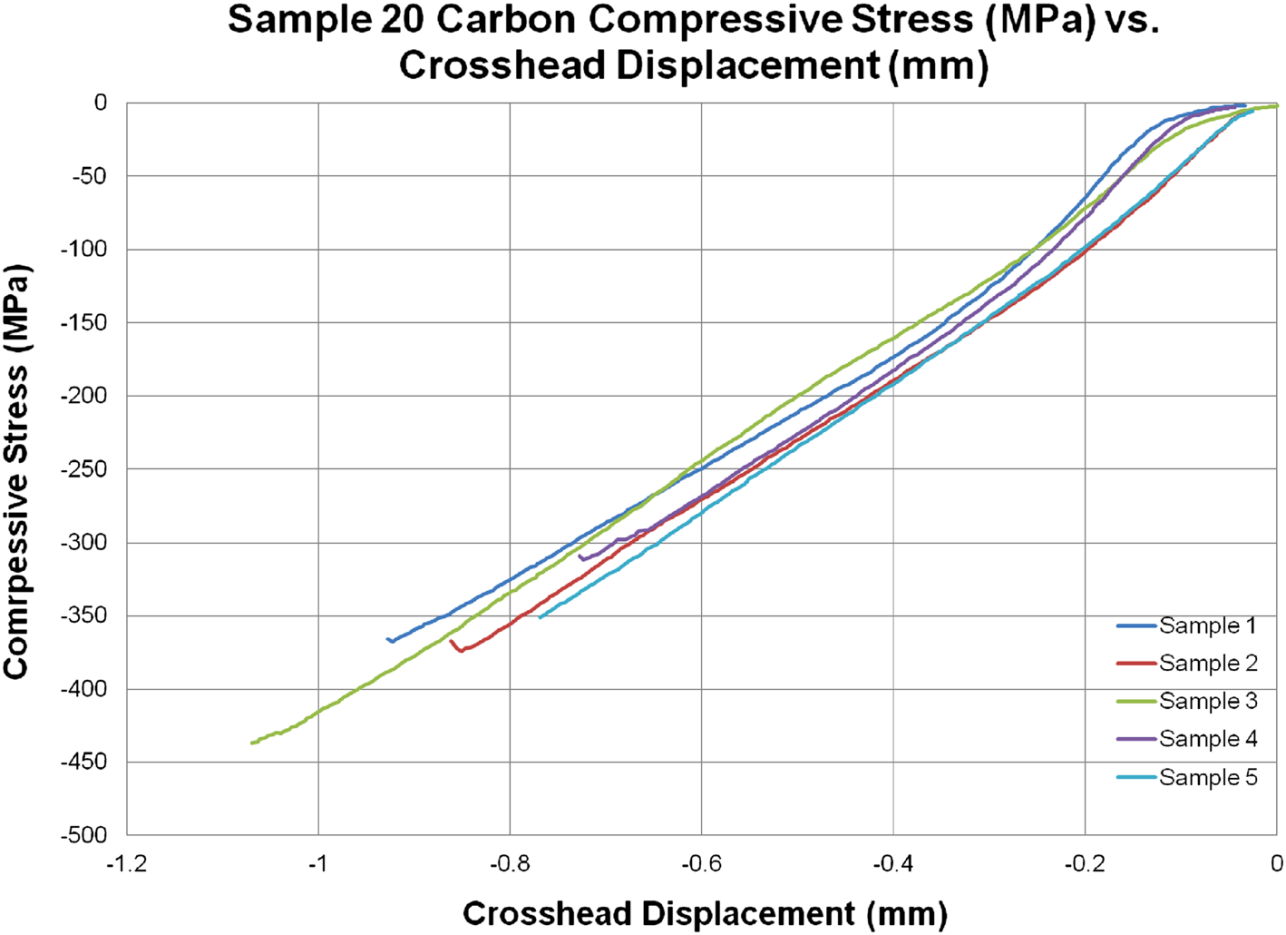
Comprehensive compression stress–strain curves for 20C.

**Figure 9:**
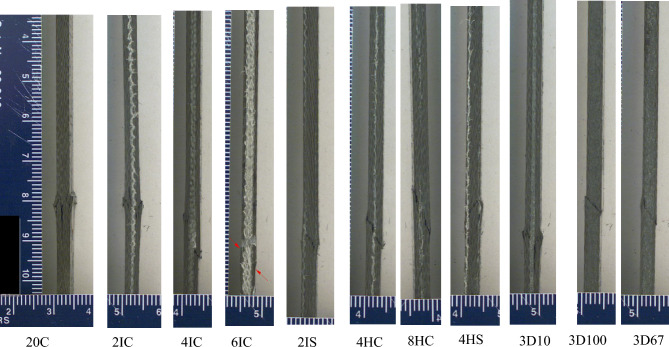
Failure modes in compression for different variants.

Table A3 and Fig. 8 provide representative stress–strain curves 20C in compression. A similar set of curves are included in Fig. A1, Appendix A for 2IC to demonstrate repeatability of compression tests. Only two curves are included in the manuscript (Fig. 8 and Fig. A1), each depicts 5 specimens tested per variant. Similar to tension and flexure response the ductility of the Innegra variants remained impressive even in case of compression loading. Figure 9 provides representative failure modes for all the variants in compression.

Compression failure was observed to be localized for all the specimens. An average 15% reduction in compression modulus and 42% reduction in compression strength was observed compared to corresponding compression modulus and strength for 20C. The failure modes for Innegra variants were less clear in the Innegra constituent, however, microcracking in the carbon layers was observed for most of the variants as seen in Fig. 9. Interestingly the compression cracks were longer and connected through the thickness for the intra-ply hybrid variants—4HC, 8HC, 8HS. It can be argued that carbon fibers within the intra-ply hybrids continue to provide a failure path (due to lower strain of the carbon fiber). However, for all inter-ply Innegra variants (2IC-6IC), 2IS, the compression shear cracks stopped at the interface between the carbon and Innegra layers. This suggests that Innegra helps with crack arrest. The 3DEP variants 3D10, 3D67 and 3D100 had clear compression shear fracture in the carbon constituents.

For 2IC, 13% reduction in compression modulus and 42% reduction in compression strength was noted when compared to compression values for 20C. Variants with higher content of Innegra for example the 6IC exhibited 65% lower strength and 56% lower modulus in comparison to 20C in compression.

#### 4HS vs 4HC

The hybrid intra-ply systems 4HS and 4HC performed well in compression compared to inter-ply laminates (such as 2IC, 4IC, 6IC, 2IS etc.). The modulus reduction was only in the 7–13% range and the strength reduction was 22–27% in comparison to 20C. However, for higher hybrid content, i.e., 8HC, reduced the compression modulus (by 25%) and strength (by 16%) compared to 4HC.

The 3DEP laminates exhibited the expected modulus benefits from carbon fiber. While there was no clear trend between 3D/10, 3D/67 and 3D/100 it can be noted that the modulus and strength were influenced by onset and progression of microcracks within the discontinuous fiber which are statistical in nature. Similar response was noted in the tensile testing of the 3DEP variants.

#### Discussion

It is seen that the strength reduces significantly in compression (by about 40% or greater), while the modulus also reduces in the order of ~ 15%. This suggests that the local microbuckling is a dominant failure mode. Further the Innegra-carbon layer variants have low interfacial bonding of the polypropylene to carbon fiber. For the 4HC versus 8HC, the properties for the 8HC were lower i.e. 25% lower strength and 16% lower modulus respectively. In all these cases the Innegra laminates were ~ 60% or higher in strain to failure indicating the significant ductility from the Innegra fibers. None of the failures were catastrophic. The weak interface between the Innegra and the carbon fiber was the primary cause for failure initiation.

The 3DEP variants were clearly influenced by the discontinuous fiber to Innegra interface. Since there was no clear trend between the 3D/10, 3D/67 and 3D/100% carbon fiber content, this points to the inherent randomly oriented fibers in the 3DP composites. The variations in the 3DEP were higher than in the standard woven carbon fabric.

### In-plane shear testing (IPSS)

**Figure 10:**
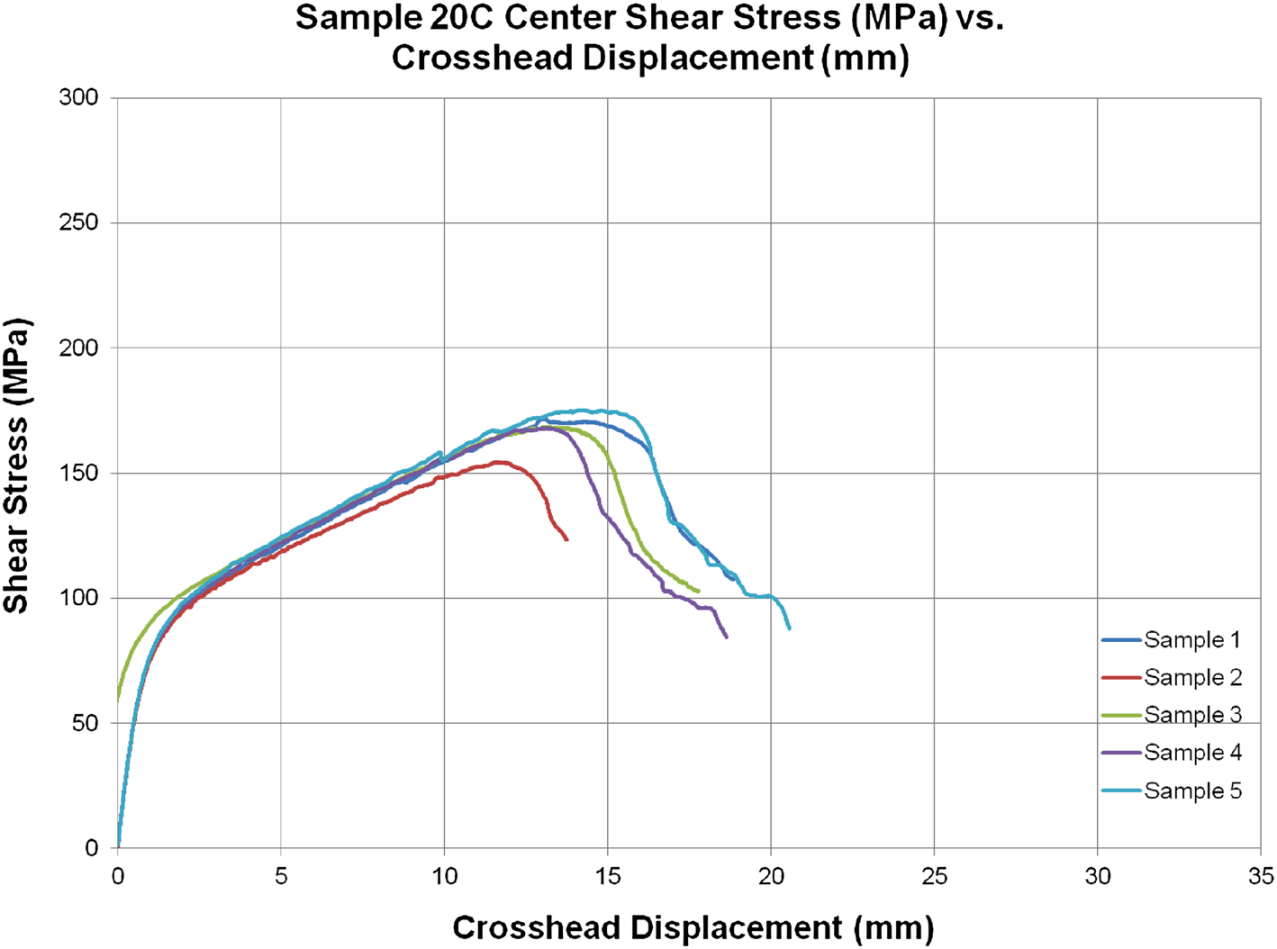
Representative stress–strain curves for in-plane shear testing for 20C.

**Figure 11:**
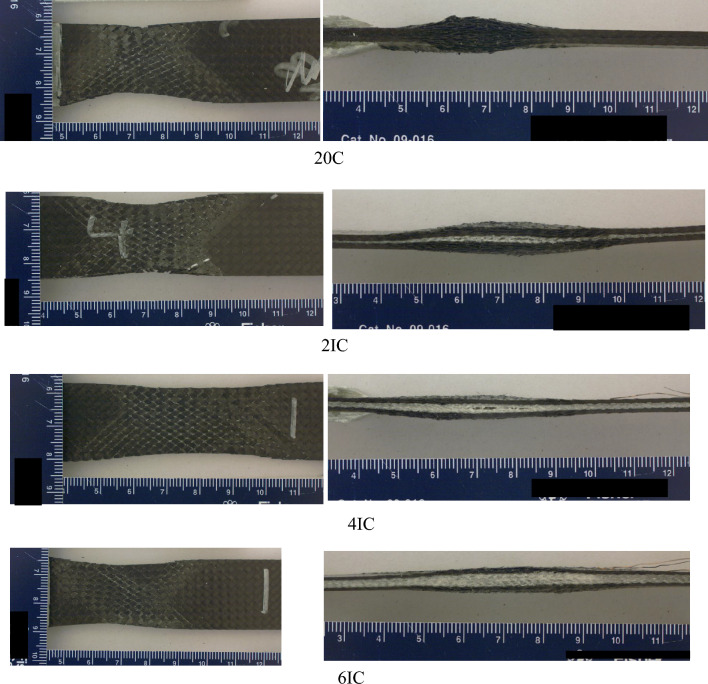
Failure mode for in-plane shear testing for different variants. The entire set of failure modes are provided in Appendix A.

Table A4 and Fig. 10 provides representative stress-train curves for the variants tested for in-plane shear testing. The stress–strain response is highly non-linear due to the off-axis deformation of the plies. Figure 10 represents repeatability for 5 specimens tested and similar curves were generated for all variants. The in-plane shear modulus of the 3DEP, i.e. 3D/100 and 3D/67 was 40% higher and the in-plane shear strength was 11% higher compared to 20C respectively. Innegra variants such as 4IC, 6IC were lower in terms of IPSS, however hybrid variants 4HC and 4HS were comparable to each other, only lower by 12% strength and 18% in modulus compared to the 20C baseline. Figure 11 provides detailed failure mode for representative variants. Figure A2 includes a comprehensive set of failure modes that provide valuable insight in terms of the role of Innegra-carbon layer interfaces.

#### Discussion

To recap, in tension and compression tests, the strength differences of the Innegra variants is greater than the modulus when compared to 20C. The significant shear deformation is evident from the stress–strain curves (Fig. 10) in all the variants (also see Fig. 11). The intra-ply variants 4HS and 8HC have lower in-plane distortion compared to other variants. The progression of failure in these was via significant distortion of the Innegra and final debonding from the carbon fiber layers. The 3D67 and 3D100 showed least amount of shear distortion primarily attributed to the randomness of the fibers in these variants. However, for IPSS, the modulus differences in the Innegra variants were greater than the strength. This suggests that due to the effective ductility of the Innegra in the ± 45 degree, it begins to deform under in-plane loading. It is less dependent on the interface, i.e. the failure is not dominated by the Innegra-carbon layer interface, but is influenced more by the deformation of the Innegra under ± 45 loading direction. This can be seen for images of all Innegra variants in Fig. 11. The highest IPSS was obtained from 3DEP variants 3D/100 and 3D/67. The in-plane shear strength is higher due to the three-dimensional distribution of fibers in the 3DEP, providing high resistance to in-plane shear.

### Low velocity impact testing

**Figure 12:**
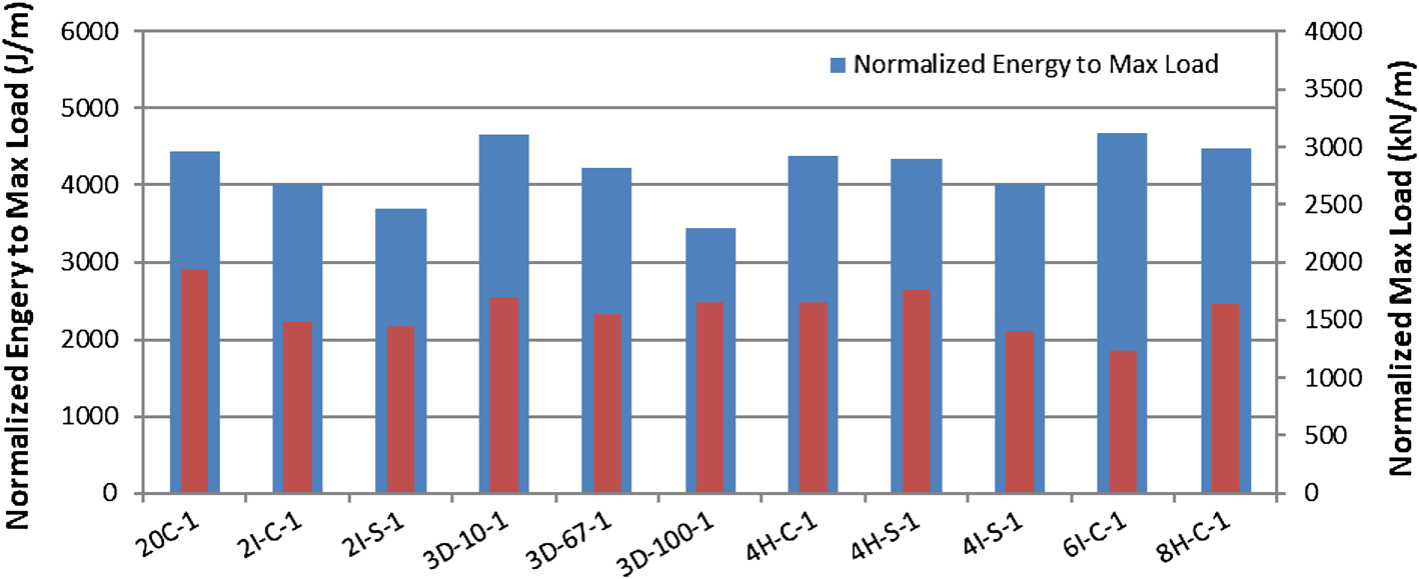
Low velocity impact testing at 15 J, Impactor: 5/8″ hemispherical shape And Impact mass: 13.6 lbs (6.15 kg).

**Figure 13:**
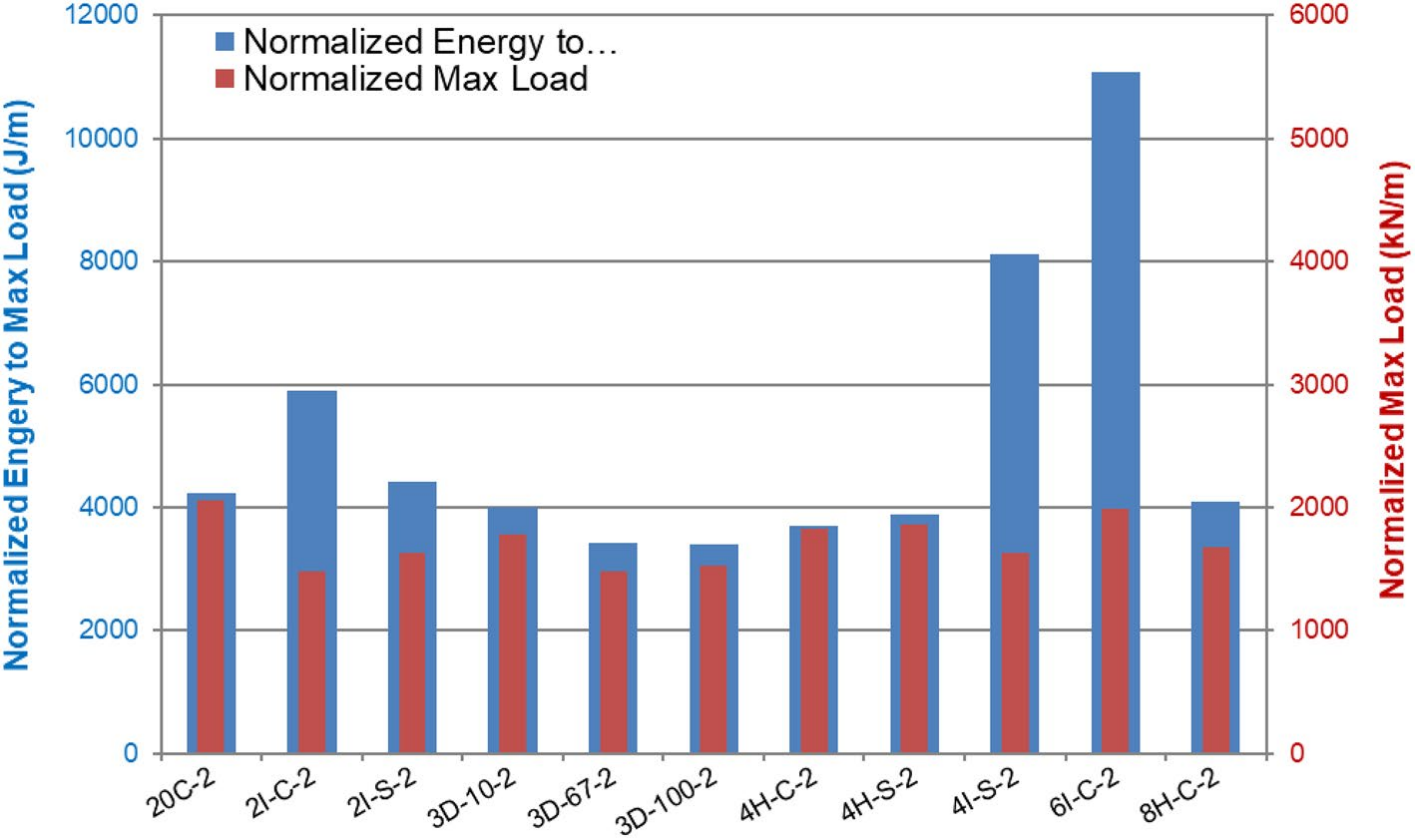
Low velocity impact testing at 60 J, Impactor: 5/8″ hemispherical shape and Impact mass: 13.6 lbs (6.15 kg).

**Figure 14:**
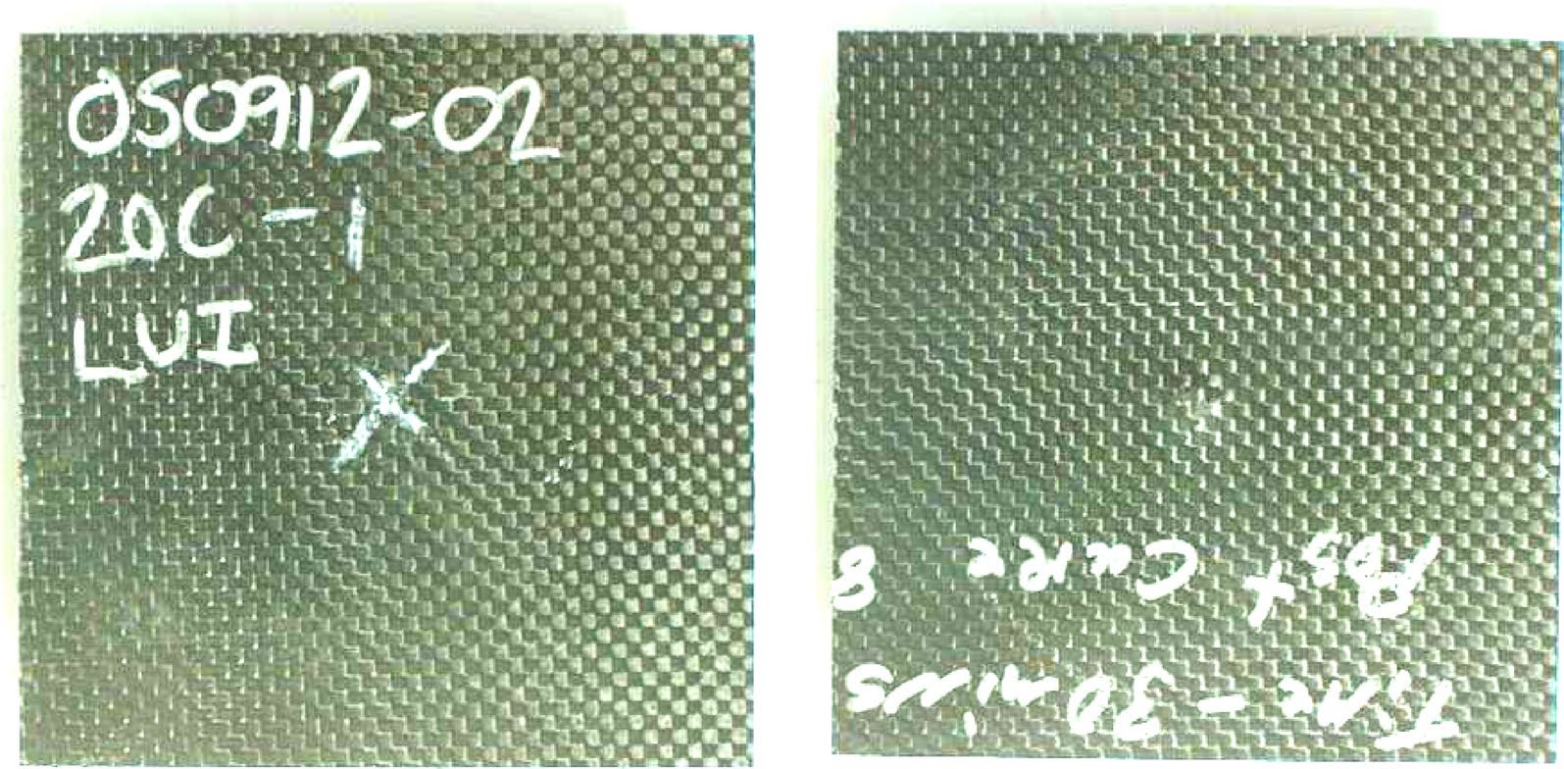
Representative 15 J low velocity impact to panel 20C. There was no notable visible front or back face damage to the panel. Other variants had very similar response and hence 15 J images are not included expect for these as representative.

**Figure 15:**
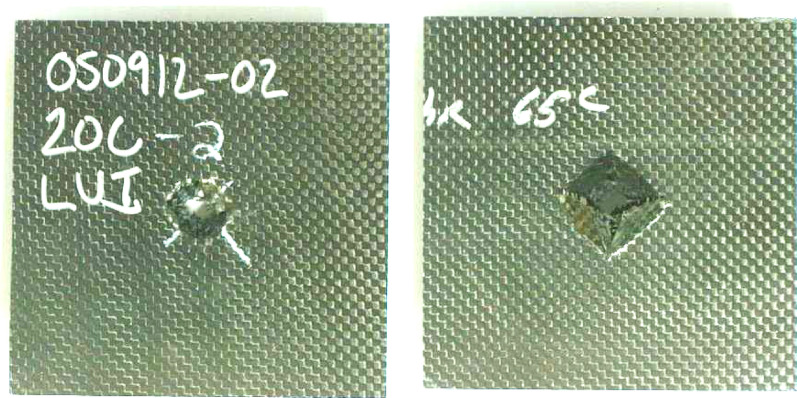
20C—Representative 60 J impact to panel 20C. The damage is highly localized, and the same phenomenon was observed for all variants. Hence, the remaining images are show as close-up of the failure location for clear view of the front and back failure local failure.

Tables A5 and A6 summarize the low velocity impact tests at 15 J and 60 J impact energies respectively. Figures 12 and 13 compare the normalized maximum load and normalized maximum energy absorbed for these energies respectively. Figures 14 and 15 provides the front and back face impact to specimen 20C at 15 J and 60 J respectively. Several of these are included in Appendix A, see Fig. A3. These are full plate images to illustrate localized failure. From Fig. 14 it can be seen that 15 J impact imparts minimal damage to the 20C plate and the damage is barely visible on the front and back end respectively. This was the case for most specimens, hence images for 15 J impacts are not provided for the remaining variants. However, from Fig. 15 it can be seen that for 60 J impact, 20C shows clear localized penetration of the impact face, and a crosshair damage on the back face. The back face damage is from tensile fracture of the carbon fibers. Figure A3 provides a range of front and back face damage for all the variants, with images zoomed at the impact damage site.

The damage modes are almost identical for all the variants as seen in Figs. 14 and 15. At 60 J the impact face witnesses penetration and the back face indicates tensile fracture in a crosshair pattern. The force–time history and energy absorbed are a function of the resistance to penetration of the impactor. The highest penetration resistance was exhibited by 2IC, 4IC and 6IC respectively. All these variants are inter-ply hybrid laminates with high Innegra content. The intra-ply variants (4HC, 8HC) had lower impact energy absorption than the inter-ply indicating that the carbon in the intra-ply configuration constraints the layer from deforming, and hence lowers its impact resistance. The 3DEP variants had clean front face penetration (less resistance) and perforation (clean back face penetration) indicative of low energy absorption.

#### Discussion

##### Low impact energy (15 J)

At lower impact energy (15 J), the differences in energy absorption across the variants was not clearly discernable; they all fell within 25% of each other in terms of normalized impact energy absorption. The lowest value was from 3D/100 (complete penetration failure) and the highest values were from 3D/10 and 6IC respectively.

##### High impact energy 60 J

The impact energy benefits of Innegra was best seen for the high energy impacts i.e. 60 J. The inter-ply variants—2IC, 4IC and 6IC outperformed all other variants. When compared to the 20C baseline, the normalized impact energy of 2IC, 4IC and 6IC was higher by 40%, 50% and 60% respectively. This suggests that when Innegra is subjected to high energy transverse impact, the fibers absorb energy by stretching as evident from the results. It may be noted that the normalized maximum load was still highest for 20C (stiffest), and the other variants were less than those for 20C within 28% of each other in terms of lower–upper bounds. The lowest normalized peak load was recorded for 3D/67.

The intra-ply hybrids, 4HC, 4HS, 8HS did not function as effectively when compared to the inter-ply hybrids, 2IC, 4IC, 6IC respectively. This can be interpreted that the carbon tows within the weave (hybrid intra-ply weave) constrain the Innegra from stretching during impact and do not contribute effectively to impact, Layered Innegra (inter-ply) with layered carbon as individual constituents seem to do better under impact.

The 3DEP variants did not perform well in impact. The layup was prone to microcracking, and the discontinuous fibers breached readily. Even at 15 J, the 3D/100 for example reached full penetration at maximum normalized load of 1500 kN/m.

## Conclusions

The study demonstrated material synergy between high stiffness carbon fiber and ductile high strength Innegra (polypropylene) fiber in different layups. Innegra complements both continuous and discontinuous carbon fiber to provide ductility and crack arrest in the composite.

Hybridization of carbon fiber with Innegra reduced strength and modulus (tensile, compression, flexure and in-plane) on an average of 18–20%, however increased the strain-to-failure (ductility) by > 20%. Progressive failure mode was observed for all Innegra variants indicating superior damage tolerance.

The position of Innegra layers in the laminate had considerable influence on the performance of the laminate. Placing Innegra towards the outer layers provided ~ 20% higher tensile strength and modulus. The constraint offered by the carbon fiber to the Innegra plies leads to onset and progression of debonding.

Compression failure was localized in all variants. The compression failure was arrested at the interface between Innegra and carbon fiber. Higher percent of Innegra adversely affected compression properties. Inter-ply hybridization was effective in blunting the crack propagation in carbon layers.

The primary mode of damage in all loading cases was through onset of debonding and propagation at the Innegra-carbon fiber interface(s). The interface of the Innegra-carbon plays a key role and in this study all cracks stopped at the Innegra-carbon interface. This influence was more prominent in tension and compression, less pronounced in flexure and impact. The constraining effect of carbon fiber was evident in the in-plane shear tests.

Hybrid Innegra-carbon exhibited enhanced energy absorption for both inter-ply and intra-ply configurations compared to carbon only. Hybrid intra-ply Innegra-carbon provided minimal benefits in terms of energy absorption under high level impact. The impact benefits were best realized in inter-ply from constituent layering of Innegra with carbon fibers. At low impact energy (15 J) the benefits of Innegra-carbon were not fully realized, however the Innegra-carbon exhibited superior damage tolerance at high energy impact (60 J). The inter-ply Innegra-carbon had higher performance than intra-ply hybrid laminates.

The 3DEP laminates exhibited higher properties in in-plane shear due to the three-dimensional interactions of the Innegra-carbon fiber network. The discontinuous 3DEP material performed best in in-plane shear, the reasoning was thought to be three-dimensional network of fibers that resist shear, while the lamina within carbon or carbon/Innegra is prone to in-plane shear. This work is of value to designers/engineers considering use of recyclable self-reinforced polypropylene in conjunction with high performing carbon fiber.

### Supplementary Information

Supplementary Information.

## Data Availability

All data generated or analyzed during this study are included in this published article [and its supplementary information files.
