# Supplementary material for: Performance of hybrid Innegra-carbon fiber composites
Source: Sci Rep. 2023 Nov 27;13:20876. doi: 10.1038/s41598-023-47353-9 (PMC10682129; doi:10.1038/s41598-023-47353-9)
Supplement: Supplementary file 1 — Supplementary Information. [file 41598_2023_47353_MOESM1_ESM.docx]

Supplemental Information (SI)

SI Table 1. Summary of tensile test results for the Innegra S, Woven Carbon and 3DEP variants

| Sequence | Nomenclature | Architecture | Average Tensile Modulus | Average Tensile Strength | % Modulus reduction relative to 20C | %Strength reduction relative to 20C |
| --- | --- | --- | --- | --- | --- | --- |
|  |  |  | GPa | MPa |  |  |
| 1 | 20C | C-C-C-C-C-C-C-C-C-C-C-C-C-C-C-C-C-C-C-C | 76.90 | 642.0 | 0 | 0 |
| 2 | 2IC | C-C-C-C-C-C-C-C-I-I-C-C-C-C-C-C-C-C | 62.40 | 518.0 | 0.18 | 0.19 |
| 3 | 4IC | C-C-C-C-C-C-I-I-I-I-C-C-C-C-C-C | 46.50 | 477.0 | 0.39 | 0.25 |
| 4 | 6IC | C-C-C-C-I-I-I-I-I-I-C-C-C-C | 33.20 | 343.0 | 0.56 | 0.46 |
| 5 | 2IS | C-C-I-C-C-C-C-C-C-C-C-C-C-C-C-I-C-C | 60.00 | 608.0 | 0.21 | 0.05 |
| 6 | 4HC | C-C-C-C-C-C-C-C-H-H-H-H-C-C-C-C-C-C-C-C | 65.20 | 327.0 | 0.02 | 0.49 |
| 7 | 8HC | C-C-C-C-C-C-H-H-H-H-H-H-H-H-C-C-C-C-C-C | 57.80 | 549.0 | 0.24 | 0.14 |
| 8 | 4HS | C-C-H-H-C-C-C-C-C-C-C-C-C-C-C-C-H-H-C-C | 65.20 | 618.0 | 0.15 | 0.03 |
| 9 | 3D/10 | C-C-C-C-C-C-C-C-[3D/10]-[3D/10]-C-C-C-C-C-C-C-C | 58.20 | 580.0 | 0.24 | 0.09 |
| 10 | 3D/100 | C-C-C-C-[3D/100]-[3D/100]-C-C-C-C | 50.90 | 455.0 | 0.33 | 0.29 |
| 11 | 3D/67 | C-C-C-C-[3D/67]-[3D/67]-C-C-C-C | 43.20 | 437.0 | 0.43 | 0.32 |

SI Table 2. Summary of flexural test results for the Innegra S, Woven Carbon and 3DEP variants

| Sequence | Nomenclature | Architecture | Average Flex Modulus | Average Flex Strength | % Reduction in flexural modulus compared to 20C | % Reduction in flexural strength compared to 20C |
| --- | --- | --- | --- | --- | --- | --- |
|  |  |  | GPa | MPa |  |  |
| 1 | 20C | C-C-C-C-C-C-C-C-C-C-C-C-C-C-C-C-C-C-C-C | 54.60 | 638.0 | 0 | 0 |
| 2 | 2IC | C-C-C-C-C-C-C-C-I-I-C-C-C-C-C-C-C-C | 27.60 | 564.0 | 0.49 | 0.12 |
| 3 | 4IC | C-C-C-C-C-C-I-I-I-I-C-C-C-C-C-C | 21.50 | 258.0 | 0.60 | 0.59 |
| 4 | 6IC | C-C-C-C-I-I-I-I-I-I-C-C-C-C | 20.90 | 213.0 | 0.61 | 0.66 |
| 5 | 2IS | C-C-I-C-C-C-C-C-C-C-C-C-C-C-C-I-C-C | 33.10 | 388.0 | 0.39 | 0.39 |
| 6 | 4HC | C-C-C-C-C-C-C-C-H-H-H-H-C-C-C-C-C-C-C-C | 44.40 | 413.0 | 0.18 | 0.35 |
| 7 | 8HC | C-C-C-C-C-C-H-H-H-H-H-H-H-H-C-C-C-C-C-C | 36.60 | 344.0 | 0.33 | 0.46 |
| 8 | 4HS | C-C-H-H-C-C-C-C-C-C-C-C-C-C-C-C-H-H-C-C | 42.10 | 465.0 | 0.23 | 0.27 |
| 9 | 3D/10 | C-C-C-C-C-C-C-C-[3D/10]-[3D/10]-C-C-C-C-C-C-C-C | 45.10 | 566.0 | 0.17 | 0.11 |
| 10 | 3D/100 | C-C-C-C-[3D/100]-[3D/100]-C-C-C-C | 46.70 | 523.0 | 0.14 | 0.18 |
| 11 | 3D/67 | C-C-C-C-[3D/67]-[3D/67]-C-C-C-C | 43.00 | 498.0 | 0.21 | 0.22 |

SI Table 3. Summary of compression test results for the Innegra, Woven Carbon and 3DEP variants

| Sequence | Nomenclature | Architecture | Average Compressive Strength | Average compressive modulus | % Reduction in compression strength modulus compared to 20C | % Reduction in compression modulus compared to 20C |
| --- | --- | --- | --- | --- | --- | --- |
|  |  |  | MPa |  |  |  |
| 1 | 20C | C-C-C-C-C-C-C-C-C-C-C-C-C-C-C-C-C-C-C-C | 368.2 | 64.18 |  |  |
| 2 | 2IC | C-C-C-C-C-C-C-C-I-I-C-C-C-C-C-C-C-C | 299.0 | 54.00 | 0.187 | 0.158 |
| 3 | 4IC | C-C-C-C-C-C-I-I-I-I-C-C-C-C-C-C | 202.8 | 42.35 | 0.45 | 0.34 |
| 4 | 6IC | C-C-C-C-I-I-I-I-I-I-C-C-C-C | 126.9 | 28.12 | 0.65 | 0.56 |
| 5 | 2IS | C-C-I-C-C-C-C-C-C-C-C-C-C-C-C-I-C-C | 256.9 | 54.49 | 0.22 | 0.15 |
| 6 | 4HC | C-C-C-C-C-C-C-C-H-H-H-H-C-C-C-C-C-C-C-C | 286.0 | 59.20 | 0.22 | 0.07 |
| 7 | 8HC | C-C-C-C-C-C-H-H-H-H-H-H-H-H-C-C-C-C-C-C | 214.5 | 49.65 | 0.42 | 0.22 |
| 8 | 4HS | C-C-H-H-C-C-C-C-C-C-C-C-C-C-C-C-H-H-C-C | 267.7 | 55.27 | 0.27 | 0.14 |
| 9 | 3D/10 | C-C-C-C-C-C-C-C-[3D/10]-[3D/10]-C-C-C-C-C-C-C-C | 269.3 | 53.88 | 0.26 | 0.16 |
| 10 | 3D/100 | C-C-C-C-[3D/100]-[3D/100]-C-C-C-C | 268.7 | 43.39 | 0.27 | 0.32 |
| 11 | 3D/67 | C-C-C-C-[3D/67]-[3D/67]-C-C-C-C | 202.0 | 38.15 | 0.45 | 0.405 |

SI Figure 1. Compression curves for sample 2I representing repeatability from 5 specimens

SI Table 4. Summary of in-plane tensile shear strength and modulus results for the Innegra S, Woven Carbon and 3DEP variants

| **Sequence** | **Nomenclature** | **Architecture** | **Average Shear**  **Strength** | **Average Shear**  **Modulus** | **Average Shear**  **Poisson Ratio** |
| --- | --- | --- | --- | --- | --- |
|  |  |  | **MPa** | **GPa** |  |
| 1 | 20C | C-C-C-C-C-C-C-C-C-C-C-C-C-C-C-C-C-C-C-C | 85.82 | 10.24 | 0.73 |
| 2 | 2IC | C-C-C-C-C-C-C-C-I-I-C-C-C-C-C-C-C-C | 78.98 | 8.44 | 0.73 |
| 3 | 4IC | C-C-C-C-C-C-I-I-I-I-C-C-C-C-C-C | 60.56 | 5.96 | 0.71 |
| 4 | 6IC | C-C-C-C-I-I-I-I-I-I-C-C-C-C | 46.05 | 4.33 | 0.70 |
| 5 | 2IS | C-C-I-C-C-C-C-C-C-C-C-C-C-C-C-I-C-C | 75.40 | 8.30 | 0.70 |
| 6 | 4HC | C-C-C-C-C-C-C-C-H-H-H-H-C-C-C-C-C-C-C-C | 75.08 | 8.32 | 0.69 |
| 7 | 8HC | C-C-C-C-C-C-H-H-H-H-H-H-H-H-C-C-C-C-C-C | 63.82 | 4.59 | 0.73 |
| 8 | 4HS | C-C-H-H-C-C-C-C-C-C-C-C-C-C-C-C-H-H-C-C | 76.15 | 10.04 | 0.69 |
| 9 | 3D/10 | C-C-C-C-C-C-C-C-[3D/10]-[3D/10]-C-C-C-C-C-C-C-C | 77.57 | 9.50 | 0.68 |
| 10 | 3D/100 | C-C-C-C-[3D/100]-[3D/100]-C-C-C-C | 120.34 | 19.72 | 0.50 |
| 11 | 3D/67 | C-C-C-C-[3D/67]-[3D/67]-C-C-C-C | 93.80 | 14.87 | 0.50 |

2IS

4HC

8HC

4HS

SI Figure 2. Representative failure modes for in-plane shear of different variants. Some failure modes were shown in the main body of the manuscript

3D10

3D100

3D67

SI Table 5. Summary of low velocity impact tests at 15 J for the Innegra, Woven Carbon and 3DEP variants

| Sample | Velocity (m/s) | Energy to max load (J) | Maximum load (kN) | Thickness (mm) | E/t (J/m) | F/t (kN/m) |
| --- | --- | --- | --- | --- | --- | --- |
| 20C-1 | 2.22 | 14.38 | 6.31 | 3.24 | 4436.91 | 1946.05 |
| 2I-C-1 | 2.22 | 12.93 | 4.79 | 3.22 | 4016.43 | 1486.74 |
| 2I-S-1 | 2.22 | 11.83 | 4.65 | 3.21 | 3686.20 | 1449.91 |
| 3D-10-1 | 2.23 | 15.13 | 5.50 | 3.25 | 4655.35 | 1692.58 |
| 3D-67-1 | 2.22 | 13.98 | 5.13 | 3.31 | 4223.63 | 1548.61 |
| 3D-100-1 | 2.22 | 11.40 | 5.47 | 3.32 | 3434.55 | 1647.41 |
| 4H-C-1 | 2.22 | 14.25 | 5.38 | 3.25 | 4386.06 | 1653.88 |
| 4H-S-1 | 2.22 | 14.10 | 5.70 | 3.25 | 4339.97 | 1754.68 |
| 4I-S-1 | 2.23 | 12.89 | 4.54 | 3.22 | 4003.60 | 1410.62 |
| 6I-C-1 | 2.22 | 15.12 | 3.97 | 3.23 | 4681.83 | 1230.53 |
| 8H-C-1 | 2.22 | 14.59 | 5.34 | 3.26 | 4474.17 | 1639.17 |

2IC 4IS

4IC 2IS

4HC 8HC

4HS 3D10

3D67 3D100

SI Figure 3. Failure mode under 15 J and 60 J impact energy respectively

SI Table 6. Summary of low velocity impact tests at 60 J for the Innegra, Woven Carbon and 3DEP variants

| Sample | Velocity (m/s) | Energy to max load (J) | Maximum load (kN) | Thickness (mm) | E/t (J/m) | F/t (kN/m) |
| --- | --- | --- | --- | --- | --- | --- |
| 20C-2 | 4.42 | 13.93 | 6.77 | 3.29 | 4233.10 | 2056.50 |
| 2I-C-2 | 4.41 | 18.95 | 4.75 | 3.21 | 5903.24 | 1479.00 |
| 2I-S-2 | 4.42 | 14.24 | 5.25 | 3.22 | 4421.34 | 1629.41 |
| 3D-10-2 | 4.42 | 13.02 | 5.79 | 3.26 | 3992.52 | 1777.55 |
| 3D-67-2 | 4.42 | 11.14 | 4.79 | 3.25 | 3426.74 | 1474.62 |
| 3D-100-2 | 4.42 | 11.21 | 5.04 | 3.3 | 3397.12 | 1527.55 |
| 4H-C-2 | 4.42 | 11.98 | 5.90 | 3.23 | 3709.16 | 1827.21 |
| 4H-S-2 | 4.42 | 12.54 | 5.99 | 3.23 | 3881.67 | 1855.51 |
| 4I-S-2 | 4.42 | 25.99 | 5.22 | 3.2 | 8121.50 | 1631.34 |
| 6I-C-2 | 4.41 | 35.10 | 6.29 | 3.17 | 11072.08 | 1985.52 |
| 8H-C-2 | 4.42 | 13.39 | 5.49 | 3.27 | 4096.02 | 1680.37 |
